# Systemic inflammatory profile and response to anti-tumor necrosis factor therapy in chronic obstructive pulmonary disease

**DOI:** 10.1186/1465-9921-13-12

**Published:** 2012-02-02

**Authors:** Matthew J Loza, Rosemary Watt, Frédéric Baribaud, Elliot S Barnathan, Stephen I Rennard

**Affiliations:** 1 Immunology Biomarkers, Janssen Research & Development, LLC, Malvern, PA, USA; 2 Immunology General, Janssen Research & Development, LLC, Malvern, PA, USA; 3 Internal Medicine, University of Nebraska Medical Center, Omaha, NE, USA

**Keywords:** chronic obstructive pulmonary disease, inflammation, biological biomarkers, tumor necrosis factor-alpha, infliximab

## Abstract

**Background:**

Chronic obstructive pulmonary disease (COPD) is characterized by progressive worsening of airflow limitation associated with abnormally inflamed airways in older smokers. Despite correlative evidence for a role for tumor necrosis factor-alpha in the pathogenesis of COPD, the anti-tumor necrosis factor-alpha, infliximab did not show clinical efficacy in a double-blind, placebo-controlled, phase II clinical trial. This study sought to evaluate the systemic inflammatory profile associated with COPD and to assess the impact of tumor necrosis factor neutralization on systemic inflammation.

**Methods:**

Serum samples (n = 234) from the phase II trial were collected at baseline and after 24 weeks of placebo or infliximab. Additionally, baseline serum samples were obtained from an independent COPD cohort (n = 160) and 2 healthy control cohorts (n = 50; n = 109). Serum concentrations of a broad panel of inflammation-associated analytes were measured using a 92-analyte multiplex assay.

**Results:**

Twenty-five proteins were significantly elevated and 2 were decreased in COPD, including highly elevated CD40 ligand, brain-derived neurotrophic factor, epidermal growth factor, acute-phase proteins, and neutrophil-associated proteins. This profile was largely independent of smoking status, age, and clinical phenotype. The majority of these associations of serum analytes with COPD are novel findings. Increased serum creatine kinase-muscle/brain and myoglobin correlated modestly with decreased forced expiratory volume at 1 second, suggesting cardiac involvement. Infliximab did not affect this systemic inflammatory profile.

**Conclusions:**

A robust systemic inflammatory profile was associated with COPD. This profile was generally independent of disease severity. Because anti-tumor necrosis factor-alpha did not influence systemic inflammation, how to control the underlying pathology beyond symptom suppression remains unclear.

**Trial Registration:**

ClinicalTrials.gov, *No*.: NCT00056264.

## Background

Chronic obstructive pulmonary disease (COPD) is a complex syndrome characterized by progressive expiratory airflow loss associated with abnormal inflammation in the lungs. In addition to symptoms related to airway pathology--including cough, excessive sputum, and dyspnea--COPD has systemic manifestations, one of which may be exercise limitation related to muscle weakness [1]. Systemic inflammation has been described in COPD, including increased production of the potent inflammatory mediator tumor necrosis factor (TNF)-alpha [2-5]. Increased TNF-alpha production has also been associated with muscle loss and weakness in COPD [5-7]. Although no natural animal models of COPD exist, intraperitoneal injection of TNF-alpha in rats leads to emphysema,[8] which may resemble the apoptosis of alveolar cells observed in COPD patients with emphysema [9,10].

Because TNF-alpha inhibitors have demonstrated clinical efficacy in various chronic inflammatory disorders,[11-13] a phase II, double-blind, multicenter, placebo-controlled clinical study was performed to evaluate the safety and efficacy of infliximab (Janssen Biotech, Inc., Horsham, PA, USA), an anti-TNF-alpha monoclonal antibody, in the treatment of COPD [14]. Infliximab failed to demonstrate improvement in the primary endpoint, the Chronic Respiratory Questionnaire (CRQ) score, and in other secondary clinical outcomes after 24 weeks of treatment. Serum samples were obtained from patients at baseline and after 24 weeks of treatment.

The goal of this study was to test the hypothesis that the lack of clinical efficacy of infliximab in COPD patients was associated with a failure of infliximab to significantly impact the underlying systemic inflammation associated with COPD. From previous pharmacodynamic studies of infliximab, several serum biomarkers, including MIP-1beta and TNF-RII, were shown to be significantly decreased by anti-TNF treatment, with changes in these biomarkers correlating with clinical efficacy [15]. In addition, the general systemic inflammatory and biochemical profile associated with COPD was defined and evaluated for whether infliximab treatment could impact this broader disease-associated profile.

## Methods

### Subjects

In the phase II, double-blind, multicenter, placebo-controlled C0168T54 study (T54), COPD patients were randomized, stratified by investigational site and smoking status, to receive placebo or infliximab 3 or 5 mg/kg at weeks 0 (baseline), 2, 6, 12, 18, and 24. Detailed background and results have been reported [14]. Peripheral venous blood samples were collected in the T54 study before study agent administration at baseline and at 24 weeks [14].

Additional serum samples from patients with mild-to-severe COPD with available demographics and disease characteristics data were purchased from a commercial vendor (BioServe Biotechnologies, Ltd., Beltsville, MD, USA) and evaluated according to Global Initiative for Chronic Obstructive Lung Disease (GOLD) criteria. Serum samples from 2 sets of healthy controls were obtained from a commercial vendor (Bioreclamation, LLC, Hicksville, NY, USA). Qualification for healthy status is detailed in the online supplement (see Additional file 1).

This study was conducted according to the principles of the Declaration of Helsinki. The institutional review board for each site in the T54 study approved the protocol. All subjects provided informed written consent.

### Measurement of serum analyte concentrations

Serum samples were analyzed for the concentrations of 92 inflammation-associated proteins by Rules-Based Medicine, Inc. (now Myriad RMB, Inc., Austin, TX, USA) using their human MAP v1.6 panel of Luminex-based multiplex assays. The analytes included in the panel are listed in the online supplement Table S1 (see Additional file 2). The handling of values below reliable quantification (least detectable dose [LDD]) is described in the online supplement (see Additional file 1). The first set of healthy control samples (Ctr1) was bioanalyzed in the same batch as the T54 and BioServe samples. The second set of healthy control samples (Ctr2) was bioanalyzed independently.

### Statistical Analyses

Mann-Whitney U tests were used to compare continuous variables among 2 groups. Fisher's exact tests were used to compare dichotomous variables. Rank-based tests using Spearman's correlations were used to test for correlations among continuous variables. Significance levels were reported as p-values or false discovery rates (FDR) to control for multiple-testing inflation of false positive rate (Benjamin-Hochberg procedure). Hierarchical standard clustering analyses were performed with average linkage and a Euclidean similarity metric (ArrayStudio, OmicSoft Corp., Cary, NC, USA).

## Results

### COPD and healthy control cohorts

Before examining whether infliximab treatment was able to modify the systemic inflammatory and biochemical profile in COPD patients, this systemic profile first needed to be rigorously established in both the study population and an independent COPD population compared to healthy control populations. In the T54 study, 234 serum samples were collected at baseline, and 200 samples were collected after 24 weeks of treatment with placebo (n = 68) or infliximab 3 mg/kg (n = 64) or 5 mg/kg (n = 68) from COPD patients, most of whom had moderate-to-very severe disease (GOLD stages II-IV). One hundred sixty serum samples were obtained from BioServe from patients with mild-to-very severe COPD distributed evenly across disease severities (GOLD stages I-IV). Fifty serum samples were obtained from Ctr1, and 109 samples were obtained from Ctr2. Demographic and clinical characteristics for each cohort are reported in Table 1.

**Table 1:** Demographic and baseline clinical phenotypes in COPD and control populations

Parameter	T54 COPD^a^					BioServe COPD^b^		Healthy controls^c^		p (COPD vs healthy control)^d^	
	
	Total	Placebo	3 mg/kg	5 mg/kg	p-value	Total	p (vs T54)	Ctr1	Ctr2	Ctr1 vs T54, BS	Ctr2 vs T54, BS
**Sample size (n)**	234^e^	68	64	68		160		50	109		
											
**Gender(n: male/female)**	140/94	43/25	40/24	37/31	0.51	73/87	0.37	30/20	54/42	(1.0, 0.52)	(0.27, 0.63)
**Age (years)**	65.2 ± 8.8	65.0 ± 8.9	64.4 ± 8.2	64.9 ± 9.6	0.93	65.8 ± 11.8	0.57	38.7 ± 11.8	49.9 ± 14.5	(< 10^-4^, < 10^-4^)	(< 10^-4^, < 10^-4^)
**BMI (kg/m^2^)**	27.2 ± 6.1	28.2 ± 6.1	26.6 ± 5.4	27.0 ± 6.6	0.32	26.5 ± 7.0	0.27	27.0 ± 6.8	27.9 ± 6.9	(0.82, 0.65)	(0.46, 0.16)
											
**Race (n)**					0.46		0.036			(< 10^-4^, < 10^-4^)	(< 10^-4^, < 10^-4^)
Black	10	3	1	4		14		25	41		
Caucasian	223	64	63	64		143		5	22		
Other	1	1	0	0		3		20	12		
											
**Smoking status (n)**					0.93		0.00011			(< 10^-4^, < 10^-4^)	(< 10^-4^, < 10^-4^)
Current smoker	104	29	29	31		50		33	36		
Ex-smoker	130	39	35	37		100					
Non-smoker						9		16	24		
											
**GOLD stage (n)**					0.66		< 10^-9^	n/a	n/a	n/a	n/a
stage I	3	1	0	2		40					
stage II	74	23	21	20		40					
stage III	104	33	31	26		40					
stage IV	53	11	16	16		40					
											
**Subtype (n)**					0.58		< 10^-9^	n/a	n/a	n/a	n/a
Chronic bronchitis	54	20	11	15		41					
Chronic emphysema	95	26	28	28		80					
Both	85	22	25	25		3					
											
**FEV_1 _(% predicted)**											
Baseline	43.4 ± 16.0	45.2 ± 16.2	43.9 ± 17.5	41.8 ± 14.9	0.49	52.7 ± 25.1	0.000051	n/a	n/a	n/a	n/a
Change from baseline	-1.1 ± 6.7	-1.4 ± 7.5	0.3 ± 6.1	-1.9 ± 7.5	0.19	n/a	n/a	n/a	n/a	n/a	n/a
											
**CRQ total score**											
Baseline	80.4 ± 19.1	83.8 ± 20.1	77.1 ± 17.5	80.2 ± 19.3	0.11	n/a	n/a	n/a	n/a	n/a	n/a
Change from baseline	10.3 ± 18.6	12.3 ± 19.2	12.7 ± 22.7	15.1 ± 10.0	0.70	n/a	n/a	n/a	n/a	n/a	n/a

^a^Clinical and demographic information for the C0168T54 COPD population is presented for all patients with baseline serum analyses performed (total) and stratified by treatment group (placebo, 3 mg/kg infliximab, or 5 mg/kg infliximab) for patients who had serum samples analyzed for both baseline and week 24 timepoints (n = 200). P-values are presented for significance of differences among the treatment groups.

^b^Clinical and demographic information for the BioServe COPD population, with p-values presented for significance of differences between BioServe and T54 COPD populations.

^c^Clinical and demographic information for the healthy control populations. Ctr1, control population samples bioanalyzed in same batch as COPD samples; Ctr2, control population samples bioanalyzed independently of the COPD and Ctr1 samples.

^d^P-values for the significance of differences between the indicated control population vs COPD population (T54, BioServe).

^e^Summary statistics are sample size (n), mean ± standard deviation, or p-value.

BMI, body mass index; COPD, chronic obstructive pulmonary disease; CRQ, chronic respiratory questionnaire; FEV_1_, forced expiratory volume in 1 second; GOLD, Global Initiative for Chronic Obstructive Lung Disease; n/a, not applicable.

### Associations of baseline serum analyte levels and COPD

**Figure 1:**
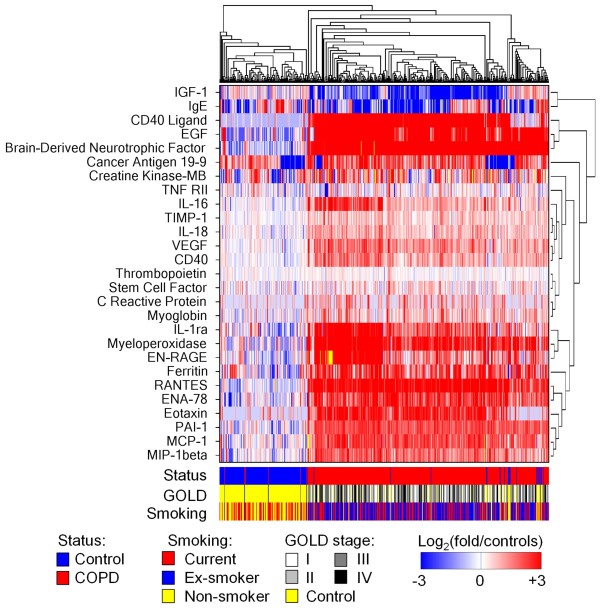
**Serum analytes associated with COPD vs controls**. Hierarchical clustering (average linkage with Euclidean distances) was performed restricted to the analytes reported in Table 2 to be associated with COPD vs controls. Serum concentrations were normalized as log_2 _of fold over the geometric mean (G_m_) of the control cohorts and presented as a heatmap with subjects across x-axis and analytes on y-axis. Disease status, smoking status, and GOLD stage are represented at bottom. IGF-1, insulin-like growth factor-1; Ig, immunoglobulin; EGF, epidermal growth factor; TNF-RII, tumor necrosis factor-receptor II; IL, interleukin; TIMP-1, tissue inhibitor of metalloproteinases-1; VEGF, vascular endothelial growth factor; EN-RAGE, extracellular newly identified-receptor for advanced glycation end-binding protein; RANTES, regulated upon activation, normally T-cell expressed, and secreted; ENA-78, epithelial-derived neutrophil activating protein-78; PAI-1, plasminogen activating factor-1; MCP-1, monocyte chemoattractant protein-1; MIP-1beta, macrophage inflammatory protein-1beta.

Differences in analyte levels were tested separately for each COPD cohort versus each control cohort for a total of 4 individual comparisons per analyte. Twenty-five serum analytes were significantly elevated by ≥ 50% (FDR < 0.05 and fold/control > 1.50) in the COPD cohorts relative to healthy controls for each of the 4 comparisons, whereas 2 analytes (insulin-like growth factor [IGF]-1 and immunoglobulin [Ig]E) were significantly lower by ≥ 50% in the COPD cohorts (Table 2, Figure 1). CD40 ligand (CD40L), epidermal growth factor (EGF), brain-derived neurotrophic factor (BDNF), Regulated upon Activation, Normally T-cell expressed, and Secreted (RANTES), and myeloperoxidase were the most highly overexpressed proteins in COPD, being on average 10-fold greater than the levels observed for the control cohorts. Potential batch effects in the independently bioanalyzed Ctr2 cohort serum samples may have impacted the results for cancer antigen (CA) 19-9 and CA125 (detailed in the online supplement - Table S2; see Additional file 3). Differences in oral and inhaled corticosteroid use did not significantly impact the observed results, as described in the online supplement (see Additional file 1). The ratio between the BioServe and T54 COPD cohorts had a geometric mean (95% CI) of 1.28 (1.15-1.42), indicating a modest bias for higher measurements in the BioServe cohort. This difference between the COPD cohorts is well below the 1.5-fold cut-off used for comparisons between COPD and control cohorts. Six analytes (CD40L, IL-16, EGF, ENRAGE, IL-1RA, and myeloperoxidase), which were among the top overexpressed analytes associated with COPD, had a median in the BioServe cohort 2-fold greater than that in the T54 cohort.

**Table 2:** Serum analytes significantly elevated in COPD

Analyte^a^		Median^b^				Fold (COPD/control)^c^				FDR^d^
			
		BS	T54	Ctr1	Ctr2	BS/Ctr1	T54/Ctr1	BS/Ctr2	T54/Ctr2	
**COPD > Control**										
CD40 Ligand	ng/mL	2.50	0.96	0.01	0.01	250	95.9	250	95.9	< 10^-8^
EGF	pg/mL	699.49	230.99	14.84	3.70	47.1	15.6	189	62.4	< 10^-8^
BDNF	ng/mL	28.50	24.80	0.52	0.56	55.0	47.9	50.5	44.0	< 10^-8^
RANTES	ng/mL	18.65	15.90	1.28	1.22	14.5	12.4	15.2	13.0	< 10^-8^
Myeloperoxidase	ng/mL	2144.71	686.50	119.47	87.50	18.0	5.7	24.5	7.8	< 10^-8^
Eotaxin	pg/mL	232.50	162.50	20.50	20.50	11.3	7.9	11.3	7.9	< 10^-8^
EN-RAGE	ng/mL	254.97	35.60	13.40	14.50	19.0	2.7	17.6	2.5	< 10^-8^
Ferritin	ng/mL	166.00	113.00	18.95	28.00	8.8	6.0	5.9	4.0	< 10^-8^
IL-1RA	pg/mL	360.50	111.50	31.82	38.00	11.3	3.5	9.5	2.9	< 10^-8^
ENA-78	ng/mL	2.07	1.68	0.32	0.35	6.5	5.2	5.9	4.8	< 10^-8^
PAI-1	ng/mL	199.00	183.50	26.05	54.00	7.6	7.0	3.7	3.4	< 10^-8^
MCP-1	pg/mL	558.00	483.00	170.00	100.00	3.3	2.8	5.6	4.8	< 10^-8^
MIP-1beta	pg/mL	341.49	259.50	99.00	87.50	3.4	2.6	3.9	3.0	< 10^-8^
Thrombopoietin	ng/mL	4.92	4.70	1.62	1.60	3.0	2.9	3.1	2.9	< 10^-8^
TIMP-1	ng/mL	211.00	192.00	70.44	67.00	3.0	2.7	3.1	2.9	< 10^-8^
IL-16	pg/mL	1174.99	440.50	233.49	273.50	5.0	1.9	4.3	1.6	< 10^-8^
VEGF	pg/mL	1275.00	933.00	447.00	383.50	2.9	2.1	3.3	2.4	< 10^-8^
Cancer antigen 19-9	U/mL	7.19	5.94	2.11	3.30	3.4	2.8	2.2	1.8	3.7 × 10^-7 ^- 4.5 × 10^-4^
CD40	ng/mL	1.84	1.06	0.61	0.59	3.0	1.7	3.1	1.8	< 10^-8^
Creatine kinase-MB	ng/mL	0.48	0.45	0.21	0.21	2.3	2.2	2.3	2.2	6.1 × 10^-7 ^- 8.3 × 10^-5^
C-reactive protein	ug/mL	4.51	3.36	2.06	1.70	2.2	1.6	2.7	2.0	4.2 × 10^-7 ^- 0.0074
Myoglobin	ng/mL	16.00	16.10	7.14	9.30	2.2	2.3	1.7	1.7	< 10^-8 ^- 3.0 × 10^-8^
Stem cell factor	pg/mL	395.00	402.00	221.00	191.00	1.8	1.8	2.1	2.1	< 10^-8^
IL-18	pg/mL	302.49	253.50	148.00	154.50	2.0	1.7	2.0	1.6	< 10^-8^
TNF-RII	pg/mL	5.85	5.18	3.38	3.20	1.7	1.5	1.8	1.6	< 10^-8^
										
**COPD < Control**										
IGF-1	ng/mL	45.30	29.30	188.55	203.00	-4.2	-6.4	-4.5	-6.9	3.0 × 10^-7 ^- < 10^-8^
IgE	ng/mL	22.85	23.25	60.89	60.00	-2.7	-2.6	-2.6	-2.6	0.0043 - 6.0 × 10^-6^

^a^Median serum concentrations of the analytes in the indicated population: T54, C0168T54 COPD; BS, BioServe COPD; Ctr1, healthy control population 1; Ctr2, healthy control population 2.

^b^Signed-fold = median in COPD/median in healthy control population. When median expression in COPD population was lower than in controls, the opposite of the reciprocal was reported.

^c^False discovery rate (calculated from Mann-Whitney p-values) for each of the 4 COPD vs control comparisons expressed as the range of FDR values.

^d^Analytes significantly associated with COPD (FDR < 0.05 and |signed-fold| > 1.5) in each of the 4 comparisons are reported, ordered by descending average fold (COPD/control) values.

BDNF, brain-derived neurotrophic factor; COPD, chronic obstructive pulmonary disease; EGF, epidermal growth factor; ENA, epithelial-derived neutrophil activating protein; EN-RAGE, extracellular newly identified-receptor for advanced glycation end-binding protein; FDR, false discovery rate; IgE, immunoglobulin E; IGF, insulin-like growth factor; IL, interleukin; -MB, -muscle/brain; MCP, monocyte chemoattractant protein; MIP, macrophage inflammatory protein; PAI, plasminogen activating factor; RA, receptor agonist; RANTES, regulated upon activation, normally T-cell expressed, and secreted; TIMP, tissue inhibitor of metalloproteinases; TNF, tumor necrosis factor; VEGF, vascular endothelial growth factor.

To assess whether the significance of the COPD-associated analytes was influenced by racial distribution, race-restricted analyses were performed, pooling the 2 COPD cohorts together and the 2 control cohorts together. When restricting the analyses to only Caucasians, all COPD-associated analytes reported in Table 2 remained significant (FDR < 0.05), except for CA19-9 (FDR = 0.40) and CRP (FDR = 0.078). Similarly, in analyses restricted to only blacks, all associated analytes retained significance, except for CA19-9 (FDR = 0.53) and IgE (FDR = 0.13). Each of the COPD-associated analytes reported in Table 2 remained significant when restricting the analyses to males or females, with only CRP in females having FDR slightly > 0.05 (online supplement -Table S3; see Additional file 4).

### Influence of smoking and age

**Figure 2:**
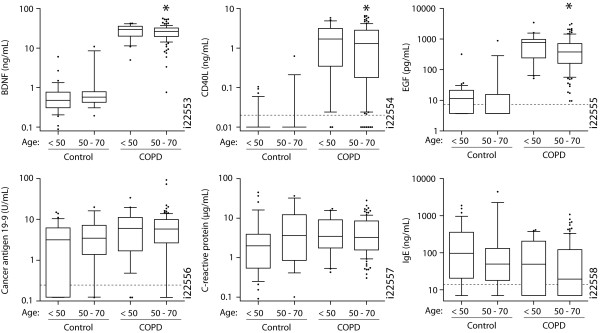
**Baseline serum analyte concentrations in current smokers**. The serum concentrations of the indicated analytes in healthy control and COPD cohorts, restricted to only current smokers, are shown for subgroups < 50 (n = 36 and 22 for controls and COPD, respectively) or between 50- to 70-years-old (n = 16 and 107 for controls and COPD, respectively). Data presented as box (median, interquartile range) and whiskers (10^th^-90^th ^percentiles). Dotted line indicates LDD. *COPD vs control, False discovery rate < 10^-8 ^within 50-70 year age group. BDNF, brain-derived neurotrophic factor; CD40L, CD40 ligand; EGF, epidermal growth factor; IgE, immunoglobulin E.

Smoking and older age together are strongly associated with risk for COPD and are therefore important to consider as confounders. When restricting comparisons to current smokers only (Figure 2, Table 3), each of the analytes significantly different between COPD and healthy controls in the unrestricted analyses remained significant for comparisons of the Ctr1 cohort to both the T54 and the BioServe COPD cohorts. For comparisons with the Ctr2 cohort, each of the analytes remained significant, except for CA19-9, CRP, IL-18, and myoglobin. Among the analytes not significantly associated with COPD in the unrestricted analyses, only carcinoembryonic antigen (CEA) was significantly elevated 60-82% in the serum samples of current smokers with COPD compared with healthy control current smokers (FDR = 1.2-5.0 × 10^-5^).

**Table 3:** Serum analytes significantly elevated in COPD among older current smokers

Analyte^a^	T54 COPD/Ctr1^b^		BioServe COPD/Ctr1		T54 COPD/Ctr2		BioServe COPD/Ctr2	
	
	Fold/control^c^	FDR^c^	Fold/control	FDR	Fold/control	FDR	Fold/control	FDR
**COPD > Control**								
CD40 Ligand	102.5	< 10^-8^	282.0	< 10^-8^	102.5	< 10^-8^	282.0	< 10^-8^
EGF	16.3	< 10^-8^	48.3	< 10^-8^	71.5	< 10^-8^	211.5	< 10^-8^
BDNF	54.0	< 10^-8^	63.9	< 10^-8^	58.5	< 10^-8^	69.3	< 10^-8^
RANTES	12.0	< 10^-8^	16.9	< 10^-8^	14.1	< 10^-8^	19.8	< 10^-8^
Myeloperoxidase	6.8	< 10^-8^	24.2	< 10^-8^	6.5	< 10^-8^	23.1	< 10^-8^
Eotaxin	8.9	< 10^-8^	10.8	< 10^-8^	8.9	< 10^-8^	10.8	< 10^-8^
EN-RAGE	3.1	< 10^-8^	25.5	< 10^-8^	2.3	1.5 × 10^-5^	19.4	< 10^-8^
Ferritin	5.6	< 10^-8^	8.7	< 10^-8^	2.9	< 10^-8^	4.5	< 10^-8^
IL-1RA	5.6	< 10^-8^	28.4	< 10^-8^	3.5	< 10^-8^	17.6	< 10^-8^
ENA-78	5.9	< 10^-8^	8.1	< 10^-8^	7.8	< 10^-8^	10.8	< 10^-8^
PAI-1	7.5	< 10^-8^	8.1	< 10^-8^	5.3	< 10^-8^	5.7	< 10^-8^
MCP-1	2.8	< 10^-8^	2.8	< 10^-8^	4.5	< 10^-8^	4.5	< 10^-8^
MIP-1beta	2.5	< 10^-8^	3.4	< 10^-8^	2.6	< 10^-8^	3.5	< 10^-8^
Thrombopoietin	2.9	< 10^-8^	3.1	< 10^-8^	2.9	< 10^-8^	3.1	< 10^-8^
TIMP-1	2.7	< 10^-8^	3.0	< 10^-8^	3.1	< 10^-8^	3.3	< 10^-8^
IL-16	1.8	< 10^-8^	5.7	< 10^-8^	1.7	< 10^-8^	5.4	< 10^-8^
VEGF	2.1	< 10^-8^	2.9	< 10^-8^	2.6	< 10^-8^	3.6	< 10^-8^
Cancer antigen 19-9	2.0	0.0026	2.2	0.0084	1.6	**0.18**	1.8	**0.25**
CD40	1.7	< 10^-8^	3.1	< 10^-8^	1.7	< 10^-8^	3.2	< 10^-8^
Creatine kinase-MB	2.1	0.0023	2.3	0.0006	2.1	0.044	2.3	0.0063
C-reactive protein	1.7	0.019	2.0	0.0076	1.7	**0.077**	2.0	0.036
Myoglobin	2.1	9.2 × 10^-6^	1.8	0.0011	1.4	0.0023	1.2	**0.074**
Stem cell factor	1.7	< 10^-8^	1.8	< 10^-8^	1.9	< 10^-8^	2.1	< 10^-8^
IL-18	1.7	< 10^-8^	2.4	< 10^-8^	1.4	4.5 × 10^-6^	2.0	< 10^-8^
TNF-RII	1.4	< 10^-8^	1.7	< 10^-8^	1.5	< 10^-8^	1.8	< 10^-8^
								
**COPD < Control**								
IGF-1	-6.0	0.00018	-3.8	6.5 × 10^-6^	-6.6	8.6 × 10^-6^	-4.2	< 10^-8^
IgE	-4.1	0.025	-3.2	0.019	-3.4	0.0050	-2.6	0.0068

^a^Baseline levels of analytes reported in Table 2 to be associated with COPD vs control, restricted to current smokers between the ages of 50 and 70, inclusive.

^b^The subpopulation sizes were: T54, n = 104; BioServe, n = 50; Ctr1, n = 33; Ctr2, n = 19.

^c^FDR and signed-fold levels in COPD over controls (fold/control) are provided for each subpopulation. FDR > 0.05 are shown in bold.

BDNF, brain-derived neurotrophic factor; COPD, chronic obstructive pulmonary disease; EGF, epidermal growth factor; ENA, epithelial-derived neutrophil activating protein; EN-RAGE, extracellular newly identified-receptor for advanced glycation end-binding protein; FDR, false discovery rate; IgE, immunoglobulin E; IGF, insulin-like growth factor; IL, interleukin; -MB, -muscle/brain; MCP, monocyte chemoattractant protein; MIP, macrophage inflammatory protein; PAI, plasminogen activating factor; RA, receptor agonist; RANTES, regulated upon activation, normally T-cell expressed, and secreted; TIMP, tissue inhibitor of metalloproteinases; TNF, tumor necrosis factor; VEGF, vascular endothelial growth factor.

When restricting analyses to current nonsmokers (including ex-smokers), each of the COPD-associated analytes reported in Table 2 remained significant for each of the 4 comparisons, except for CRP and IgE (data not shown). The lack of impact of smoking status on the COPD disease associations was consistent with limited differences between smokers and nonsmokers in the healthy control cohorts and current and ex-smokers in the COPD cohort (online supplement - Table S4; see Additional file 5). Only CEA was significantly elevated in current smokers within both the control and the COPD cohorts when compared with nonsmokers and ex-smokers, respectively.

Because of the potential impact of age on inflammation independent of disease, adjusting for this influence is important for interpretation of the above results. Therefore, statistical analyses were restricted to current smokers aged 50-70 years, combining the 2 COPD cohorts (n = 107) and the 2 control cohorts (n = 16). Each of the COPD-associated analytes reported in Table 2 were significant in the age- and current smoker-restricted comparisons, except for CA19-9, CRP, IgE, and TNF-receptor (R) II (Figure 2).

Considering the reduced statistical power from the smaller sample sizes for the smoking status- and age-restricted analyses, the COPD-associated analytes reported in Table 2 appear overall to be specific for COPD rather than associated with smoking and older age with the possible exceptions of CA19-9, CRP, and IgE.

### Correlation of inflammatory markers and disease phenotypes

**Figure 3:**
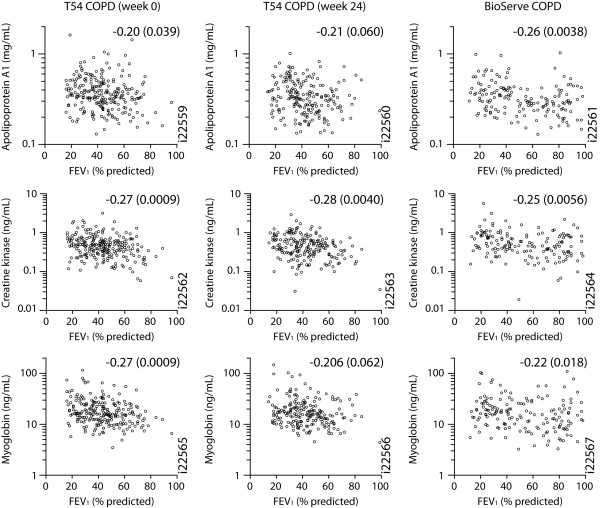
**Correlations of serum analyte concentrations and FEV_1_**. Serum concentrations of the indicated analytes (y-axes) vs percent predicted FEV_1 _(x-axes) are plotted for the T54 COPD cohort at baseline week 0 visit (left) and week 24 visit (middle) and for the BioServe COPD cohort (right). Correlation coefficients and significance of correlations are reported for each plot.

The prominent systemic inflammatory profile associated with COPD was tested for variation across disease severities and clinical phenotypes (Figure 3). Baseline serum concentrations of creatine kinase-muscle/brain (creatine kinase-MB), myoglobin, and apolipoprotein A1 were significantly (FDR < 0.05) but modestly inversely correlated with baseline percent-predicted FEV_1 _in both the BioServe and the T54 COPD cohorts (Spearman's r = -0.22 to -0.27). These correlations with FEV_1 _were also observed at week 24 in the T54 COPD cohort. Importantly, these correlations were independent of both inhaled and systemic corticosteroid usage, with no patients in the T54 COPD cohort receiving systemic steroids. Despite nominally significant higher levels of creatine kinase-MB and myoglobin in patients receiving systemic steroids, adjustment for systemic steroid use in the multivariate analyses had minimal impact on the correlations (data not shown). Likewise, restricting the analysis to patients on neither oral steroids nor inhaled steroids also had minimal impact on the correlations (data not shown).

Increased serum levels of creatine kinase-MB and myoglobin are associated with muscle tissue damage, particularly cardiac muscle damage after cardiac events. In the T54 COPD cohort, 22% of patients had a history of myocardial infarction or ischemic cardiac events; however, neither increased levels of creatine kinase-MB and myoglobin nor decreased FEV_1 _were associated with history of these cardiac events (for online supplement - Figure S1, see Additional file 6; for the figure legend, see Additional file 1).

Because of the nonstandardized methodology across clinical sites and incomplete evaluations of these COPD subcohorts with primary presentations of chronic bronchitis and chronic emphysema, the differential expression of the serum analytes between these 2 clinical phenotypes was not evaluated.

Hierarchical clustering of the T54 and the BioServe COPD cohorts was performed, restricting the analysis to analytes significantly associated with COPD as reported in Table 2 (for hierarchical clustering found in the online supplement - Figure S2, see Additional file 7; for the figure legend, see Additional file 1). The BioServe cohort was restricted to those patients with GOLD stage of ≥ II to correspond to the T54 cohort. Demographic and clinical factors, including comorbidities (data not shown), did not segregate across clusters of COPD patients. Discrete clusters of COPD patients defined by hierarchical clustering were not consistent between the 2 COPD cohorts.

### Impact of infliximab

**Figure 4:**
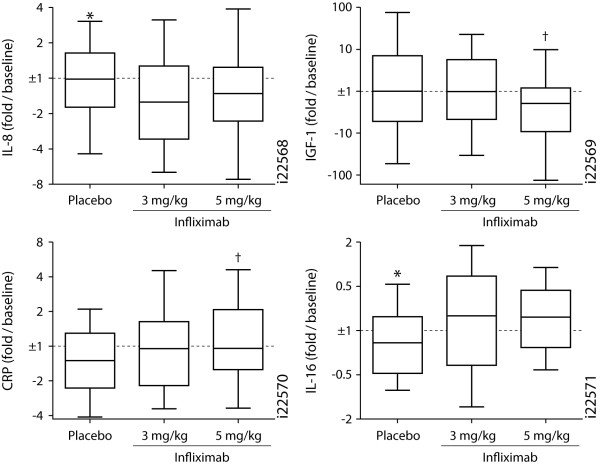
**Changes in analyte concentrations after infliximab treatment**. The serum concentrations of the indicated analytes at the 24-week timepoint for placebo and 3 and 5 mg/kg infliximab treatment groups in the T54 COPD cohort are shown as signed-fold over respective baseline concentrations. Data presented as box (median, interquartile range) and whiskers (10^th^-90^th ^percentiles). *p < 0.05 for placebo vs 3 and 5 mg/kg infliximab groups combined; † p < 0.05 for 5 mg/kg infliximab group vs placebo. IL, interleukin; IGF-1, insulin-like growth factor-1; CRP, C-reactive protein.

After establishing the systemic inflammatory and biochemical profile associated with COPD, the capacity of infliximab treatment to return this profile towards normal was examined. Changes in serum concentrations of the inflammation-associated analytes from baseline to week 24 were evaluated for the T54 COPD cohort (Figure 4). Only IL-8 (3 and 5 mg/kg infliximab groups combined) and IGF-1 (5 mg/kg infliximab group only) demonstrated trends, with high variability, for being decreased by ≥ 50% (p = 0.035 and 0.037, respectively) after infliximab treatment relative to placebo; however, IGF-1 was expressed at lower levels in COPD patients compared with controls, and IL-8 was not significantly elevated in COPD patients. Among the analytes significantly elevated in COPD patients, CRP and IL-16 showed trends for further elevation in the infliximab treatment groups (p = 0.031 and 0.0020 for the 5 mg/kg and the combined infliximab groups, respectively).

Changes in the analyte levels from baseline to week 24 also did not correlate with respective changes in FEV_1 _or CRQ total score (p > 0.05, data not shown). These results are consistent with the minimal impact of infliximab on the primary (improvement in CRQ total score) and secondary clinical outcomes (improvement in FEV_1 _and 6-minute walk distance). COPD patient subgroups expressing the highest baseline levels of CRP (top quartile, > 6.9 μg/ml) or TNF-alpha (top quartile, > 6.8 pg/ml) also did not demonstrate a significant impact of infliximab on changes in clinical and biomarker measurements (Table 4).

**Table 4:** Clinical responses to infliximab

Change from baseline^a^	Median (IQR)		p-value^b^
		
	Infliximab	Placebo	
*TNF-alpha high subset^b^*			
FEV_1_	0.0 (-4.0, 4.0)	-2.3 (-6.0, 1.0)	0.11
CRQ	8.0 (0.0, 21.5)	4.0 (-1.5, 19.0)	0.34
6MWD	0.0 (-39.0, 41.0)	21.2 (-49.4, 41.7)	0.93
Dyspnea	0.0 (0.0, 2.0)	0.0 (0.0, 2.8)	0.98
log_2_(CRP)	0.1 (-1.1, 0.9)	-0.7 (-1.5, 0.2)	0.10
			
*CRP high subset^c^*			
FEV_1_	-1.8 (-4.0, 4.0)	-2.1 (-6.0, 2.0)	0.51
CRQ	17.0 (0.0, 28.5)	4.0 (0.0, 24.0)	0.38
6MWD	3.4 (-55.6, 66.0)	0.0 (-47.7, 35.0)	0.67
Dyspnea	0.0 (-3.0, 2.0)	0.0 (0.0, 2.0)	0.40
log_2_(CRP)	-1.1 (-1.9, -0.2)	-0.4 (-1.0, 0.4)	0.075

^a^Changes from baseline in the indicated parameter in combined infliximab (3 mg/kg + 5 mg/kg) or placebo treatment, within the specified T54 COPD subpopulation.

^b^Mann-Whitney p-value for combined infliximab group vs placebo group within the indicated T54 COPD subpopulation.

^c^TNF-alpha high subset = top quartile for baseline serum TNF-alpha levels (> 6.8 pg/ml).

^d^CRP high subset = top quartile for baseline serum CRP levels (> 6.9 ug/ml).

CRP, C-reactive protein; CRQ, chronic respiratory questionnaire; FEV_1_, forced expiratory volume in 1 second; IQR, interquartile range; MWD, minute walk distance; TNF-alpha, tumor necrosis factor-alpha.

## Discussion

A novel robust systemic inflammatory profile is demonstrated here to be associated with COPD. Neutrophil-associated proteins, acute phase proteins, chemokines, and molecules associated with muscle and tissue damage were among the analytes associated with COPD. Importantly, these associations were independent of differences in current smoking status and age among the groups and were evident regardless of corticosteroid use. Despite the evident systemic inflammation, treatment with infliximab did not significantly impact the expression of these COPD-associated inflammatory markers in serum.

The associations described in this paper were derived from 2 large, independent COPD populations compared to a healthy control population. The associations were further validated in a second control population that, although bioanalyzed independently, provided a more conservative list of associations because the analytes were required to pass independently for both control populations. As presented in the online supplement -Table S5 (see Additional file 8), the majority of the COPD-associated serum analytes are novel findings, with differences between other broad-panel multiplex studies of COPD[16] in terms of lack of significance or small effects reported in those studies likely a result of the much larger COPD cohorts and well-characterized control cohorts employed in the current study.

In other diseases in which anti-TNF therapies have documented clinical efficacy, anti-TNF treatment dramatically decreased CRP levels from baseline [17-20]. Serum MIP-1beta and TNF-RII, but not CRP, were previously shown to be significantly decreased by infliximab treatment in sarcoidosis patients, with the decreases in these biomarkers correlating with the extent of clinical efficacy [15]. CRP was decreased, but only transiently, and changes in CRP levels did not correlate with clinical efficacy in that sarcoidosis study. In this study, infliximab did not significantly impact any significantly elevated COPD-associated markers in the panel, including CRP, MIP-1beta, and TNF-RII. These observations are consistent with the lack of clinical efficacy in the study. COPD patients expressing the highest baseline CRP or TNF-alpha levels also did not demonstrate a significant impact of infliximab on changes from baseline in clinical and biomarker measurements, unlike results recently reported for sarcoidosis [21]. The lack of immunologic efficacy, and perhaps clinical efficacy, may be a result of insufficient biodistribution of the drug into critical sites of pathology, namely the airways. Alternatively, the inflammation and pathology associated with COPD may be largely independent of TNF-alpha, or TNF-alpha-dependent inflammation may be already established and insensitive to subsequent TNF-alpha neutralization and its downstream effectors.

Acute-phase proteins, including CRP, extracellular newly identified-receptor for advanced glycation end-binding protein (EN-RAGE), ferritin, IL-1 receptor agonist (RA), and plasminogen activating factor (PAI)-1, were elevated in the serum samples of COPD patients. Of these, CRP [22,23] and PAI-1[24] have been previously reported to be elevated in the serum samples of COPD patients (online supplement - Table S5 [see Additional file 8]) for a summary of previously reported associations for the COPD-associated analytes listed in Table 2). IGF-1, a protein decreased during acute-phase reactions, was also lower in the serum samples of COPD patients, consistent with a previous report [25]. In nearly half of the samples in the COPD cohort, TNF-alpha levels were below LDD; however, the proportion of subjects with TNF-alpha levels above LDD was significantly higher in both COPD cohorts versus either of the 2 control cohorts (p = 0.0013 to < 10^-8^, data not shown). Although fibrinogen, another acute phase protein, has been described to be elevated in COPD, particularly in more severe disease[26], this protein was generally below detection limits in the serum samples, consistent with depletion of plasma fibrinogen during the clotting process of serum preparation.

We observed the novel result of highly elevated circulating levels of EGF in the serum samples of COPD patients, with a median of > 50-fold increase over levels in controls. EGF has been reported to be elevated in the bronchial epithelial cells of COPD patients,[27] particularly in damaged epithelia [28]. EGF can increase expression of MUC5AC,[29] a mucin associated with airway obstruction in COPD. EGF also activates fibroblasts and stimulates their proliferation;[27] however, cigarette smoke, in the absence of the clinical presentation of COPD, has been implicated in mucus production in airways via activation of the EGF receptor [30].

Other proteins significantly elevated in COPD include 6 of the 10 chemokines in the panel, T-cell/antigen-presenting cell co-stimulatory molecules (CD40, CD40L), neutrophil-associated proteins (EN-RAGE, myeloperoxidase, ENA-78), and thrombosis-related proteins (thrombopoietin, PAI-1). Other novel associations are shown in the online supplement - Table S5 (see Additional file 8).

The robust inflammatory profile associated with COPD appeared to be generally independent of disease severity (GOLD classification stage and FEV_1_). Increased inflammation has been reported in the lung tissue of patients with more severe COPD;[31] however, inflammatory markers in the peripheral blood may reflect disease activity rather than disease severity. Mannino *et al*.[32] reported in a large population-based study that elevated serum CRP concentrations are associated with COPD, with mean levels higher in patients with severe disease compared with those with mild disease. However, correlations with FEV_1 _and significance across severity levels were not formally reported. In the BioServe COPD cohort, CRP was higher in GOLD stage IV versus stage I COPD patients, consistent with that reported by Mannino *et al*. However, contrary to Mannino's findings, our data showed no difference comparing median CRP levels in GOLD stage III with stage I COPD patients (data not shown). Therefore, it is possible that CRP may either be further elevated in patients with only the most severe disease but not consistently correlated with FEV_1 _or the increased CRP levels are associated with a transient increase in disease activity (e.g., recent exacerbation) rather than severity.

The baseline inflammatory profile also did not demonstrate appreciable clustering with comorbidity history, including history of myocardial infarction and ischemic cardiac events, pneumonia, peripheral vascular disease, and diabetes (data not shown). Hierarchical clustering analysis also failed to identify discrete subpopulations of COPD patients defined by patterns of serum analyte levels that were consistent across cohorts. The lack of correlation of the extent of systemic inflammation and disease stage may suggest that a sustained systemic inflammatory state occurs early in COPD progression, perhaps before clinically-apparent onset of airway obstruction, and persists at a similar level as disease progresses despite worsening lung function.

Serum concentrations of both creatine kinase-MB and myoglobin were, however, elevated in COPD patients and inversely correlated with percent-predicted FEV_1 _in COPD patients. Increased serum levels of creatine kinase-MB and myoglobin have not been reported for stable COPD nor have correlations with FEV_1 _been observed. Increased levels of creatine kinase were reported in peripheral muscles of COPD patients [33]. Elevated levels of creatine kinase-MB and myoglobin are indicative of muscle damage and breakdown, particularly cardiac muscle, suggesting that perhaps the stress on the cardiopulmonary muscular system associated with the obstructive airway phenotype of COPD leads to the release of creatine kinase-MB and myoglobin into circulation. Consistent with a myocardial abnormality in COPD, reduced ventricular size and decreased cardiac output with normal ejection fraction consistent with diastolic dysfunction have been reported in COPD patients [34]. Importantly, creatine kinase-MB and myoglobin levels were not increased in patients with a history of myocardial infarction or ischemic cardiac events.

Smoking is a critical risk factor for COPD and has been reported to be associated with increased acute-phase protein levels in circulation, independent of COPD [35]. Although many of the smoking-elevated proteins return to normal levels after smoking cessation, some, like CRP, remain elevated for years; however, we failed to observe significant elevation of inflammatory mediators specifically in smokers without COPD. A reason for this disparity may be that the controls in this study were required to be healthy according to the rigorous entry criteria. This was consistent with other studies that we have performed comparing healthy smokers and nonsmokers (unpublished internal data, Janssen Research & Development, LLC). Conversely, in another study that included controls who were nonobstructed smokers, serum levels of inflammatory mediators were elevated to an extent observed in COPD patients. These controls, however, had some COPD-related symptoms (sputum and coughing but not dyspnea) despite not having clinically-defined airway obstruction (unpublished internal data, Janssen Research & Development, LLC).

In summary, COPD at an early stage of disease progression is associated with a robust systemic inflammatory profile independent of current smoking status. Despite the inflammation associated with COPD, including significant elevation of CRP, TNF-alpha, and other acute-phase proteins, treatment with infliximab did not significantly impact this inflammatory profile. Infliximab's lack of biochemical efficacy was consistent with its lack of clinical efficacy in COPD patients. The reason for the lack of both biochemical and clinical efficacy remains unclear, although possible explanations include TNF-alpha being a redundant contributor to inflammation in COPD and infliximab not reaching the site of local inflammation (airways) at sufficient levels to efficiently neutralize the activity of TNF-alpha.

## Competing interests

Authors Matthew J. Loza, Rosemary Watt, Frédéric Baribaud, and Elliot S. Barnathan are employees of Janssen Research & Development, LLC, Malvern, PA, USA.

## Authors' contributions

ML, EB, and RW contributed to the conception and design of study. ML performed analysis of data. ML, RW, FB, EB, and SR contributed to interpretation of data. ML drafted the article. RW, FB, EB, and SR revised it critically for important intellectual content. All authors provided final approval of the version to be published.

## Supplementary Material

Additional file 1 **Online Supplement**. Describes healthy controls exclusion criteria and serum analyte concentrations measurements in the Methods, corticosteroid influence and potential batch effects in the Results, salient References for this supplement, and the figure legends for online supplement Figures S1 and S2, found in Additional files 6 and 7. Click here for file

Additional file 2 **Online Supplement - Table S1**. Least detectable doses for analytes in Rules-Based Medicine Human MAP v1.6 panel. Analytes' least detectable doses in the Rules-Based Medicine MAP v. 1.6 panel. Click here for file

Additional file 3 **Online Supplement- Table S2**. Analytes demonstrating potential batch effects. Analytes' demonstrating potential batch effects. Click here for file

Additional file 4 **Online Supplement - Table S3**. Gender- and race-restricted analyses for associations with COPD. COPD associations with gender- and race-restricted analyses. Click here for file

Additional file 5 **Online Supplement - Table S4**. Associations of baseline analyte levels with smoking status. Baseline analyte levels and their associations with smoking status. Click here for file

Additional file 6 **Online Supplement - Figure S1**. Associations of biomarkers with history of myocardial event. Three graphs show serum levels of patients with a history of myocardial infarction or cardiac ischemia. Click here for file

Additional file 7 **Online Supplement - Figure S2**. Supervised clustering within COPD populations. Heatmap of supervised clustering within populations with COPD. Click here for file

Additional file 8 **Online Supplement - Table S5**. Previous reports for COPD-associated analytes identified herein. Compares previously reported and identified COPD-associated analytes. Click here for file
