# Supplementary material for: Systemic inflammatory profile and response to anti-tumor necrosis factor therapy in chronic obstructive pulmonary disease
Source: Respir Res. 2012 Feb 2;13(1):12. doi: 10.1186/1465-9921-13-12 (PMC3287122; doi:10.1186/1465-9921-13-12)
Supplement: Additional file 1 — Online Supplement. Describes healthy controls exclusion criteria and serum analyte concentrations measurements in the Methods, corticosteroid influence and potential batch effects in the Results, salient References for this supplement, and the figure legends for online supplement Figures S1 and S2, found in Additional files 6 and 7. [file 1465-9921-13-12-S1.DOC]

**Online Supplement**

**Systemic inflammatory profile and response to anti-tumor necrosis factor therapy in chronic obstructive pulmonary disease**

Matthew J. Loza, Rosemary Watt, Frédéric Baribaud, Elliot S. Barnathan, Stephen I. Rennard

**METHODS**

***Exclusion criteria for healthy controls***

Qualification for healthy status was evaluated from a self-report questionnaire. Exclusion criteria for healthy controls were: not feeling healthy at the time of donation; headache/fever in the past week; risky sexual activity in the past year; tested positive for or been treated for sexually transmitted diseases; history of alcoholism or drug abuse; have had ≥1 diseases in a broad panel; have been under physician care, have had surgery, or have taken prescription medications in the past year; for females, are/were pregnant in the past 6 weeks or are post-menopausal.

***Measurement of serum analyte concentrations—Least detectable dose***

Least detectable dose (LDD) was defined as the back-calculated concentration for the mean plus 3 standard deviations of luminescence values of 20 blank readings, reported in the online supplement (Table 1). Concentrations below LDD were transformed to 0.5xLDD. The 21 analytes with concentrations below LDD for >75% of baseline samples in each of the 4 cohorts were excluded from further analysis (online supplement - Table 1). The 4 analytes below LDD in ≥50% of samples in each cohort but <75% for ≥1 cohort were dichotomized as being above or below LDD for subsequent statistical analyses (online supplement - Table 1).

**RESULTS**

***Influence of corticosteroids***

Corticosteroids are anti-inflammatory mediators that can potentially affect the systemic inflammatory profile. Corticosteroids, whether inhaled or systemic (oral or intravenous), are a common therapeutic in chronic obstructive pulmonary disease (COPD). Despite the anti-inflammatory potential of corticosteroids, a strong systemic inflammatory profile was evident in both COPD cohorts. The impact of corticosteroids was anticipated to be limited in the T54 COPD cohort because oral corticosteroid treatment was not permitted from 2 weeks before screening through the end of study, except for short-term treatment of exacerbations. Oral steroid treatment, however, was permitted for the BioServe cohort. To assess the potential impact of corticosteroids in the BioServe cohort, patients not currently using corticosteroids were compared with those using inhaled but not systemic corticosteroids (data not shown). No differences were significant after multiple testing correction (FDR<0.05), except for glutathione-S-transferase (GST) in patients using inhaled corticosteroids (p=0.029, −5.0-fold). When comparing those using versus those not using systemic corticosteroids (regardless of inhaled steroid use) (data not shown), only growth hormone was decreased with nominal significance in the systemic steroid group (p=0.030, −2.8-fold). Therefore, the impact of corticosteroids on the observed associations appears to be minimal overall and across disease severities.

***Potential batch effects***

To detect potential batch effects in the independently bioanalyzed healthy control (Ctr2) cohort serum samples, significance of differences between the healthy control samples that were bioanalyzed with T54 and Bioserve samples (Ctr1) and Ctr2 cohorts for each analyte was tested. Five analytes had >50% differences with p<0.05 (online supplement - Table 2). These differences could be from batch effects in the measurements of these particular analytes or from real differences in expression levels between the 2 control cohorts. Among these analytes, only cancer antigen (CA) 19-9 demonstrated significant associations concordant for the 4 statistical comparisons. Because CA19-9 was higher in the Ctr2 cohort, the increased levels associated with COPD at most would have been modestly underestimated for the comparisons versus the Ctr2 cohort. CA125 was significant for comparisons of both COPD cohorts to the Ctr1 but not the Ctr2 cohort. Because CA125 was significantly higher in the Ctr2 cohort, the lack of significance may be a result of batch effects rather than true lack of differences. Because the comparisons of COPD versus Ctr1 were not significant for both COPD cohorts, interleukin (IL)-17, GST, and serum glutamic-oxaloacetic transaminase (SGOT) were not considered to be influenced by potential batch effects for their lack of concordant significance among comparisons.

**REFERENCES**

1. Sin DD, Man SF: **Why are patients with chronic obstructive pulmonary disease at increased risk of cardiovascular diseases? The potential role of systemic inflammation in chronic obstructive pulmonary disease**. *Circulation* 2003, **107**(11):1514-1519.

2. Piehl-Aulin K, Jones I, Lindvall B, Magnuson A, Abdel-Halim SM: **Increased serum inflammatory markers in the absence of clinical and skeletal muscle inflammation in patients with chronic obstructive pulmonary disease**. *Respiration* 2009, **78**(2):191-196.

3. Chung KF: **Cytokines in chronic obstructive pulmonary disease**. *Eur Respir J Suppl* 2001, **34**:50s-59s.

4. Papi A, Luppi F, Franco F, Fabbri LM: **Pathophysiology of exacerbations of chronic obstructive pulmonary disease**. *Proc Am Thorac Soc* 2006, **3**(3):245-251.

5. Jahnz-Ró yk K, Plusa T, Mierzejewska J: **Eotaxin in serum of patients with asthma or chronic obstructive pulmonary disease: relationship with eosinophil cationic protein and lung function**. *Mediators Inflamm* 2000, **9**(3-4):175-179.

6. D'Armiento JM, Scharf SM, Roth MD, Connett JE, Ghio A, Sternberg D, Goldin JG, Louis TA, Mao JT, O'Connor GT *et al*: **Eosinophil and T cell markers predict functional decline in COPD patients**. *Respir Res* 2009, **10**:113.

7. Miller M, Ramsdell J, Friedman PJ, Cho JY, Renvall M, Broide DH: **Computed tomographic scan-diagnosed chronic obstructive pulmonary disease-emphysema: eotaxin-1 is associated with bronchodilator response and extent of emphysema**. *J Allergy Clin Immunol* 2007, **120**(5):1118-1125.

8. Hens G, Vanaudenaerde BM, Bullens DM, Piessens M, Decramer M, Dupont LJ, Ceuppens JL, Hellings PW: **Sinonasal pathology in nonallergic asthma and COPD: 'united airway disease' beyond the scope of allergy**. *Allergy* 2008, **63**(3):261-267.

9. Fujimoto K, Yasuo M, Urushibata K, Hanaoka M, Koizumi T, Kubo K: **Airway inflammation during stable and acutely exacerbated chronic obstructive pulmonary disease**. *Eur Respir J* 2005, **25**(4):640-646.

10. Petersen AM, Penkowa M, Iversen M, Frydelund-Larsen L, Andersen JL, Mortensen J, Lange P, Pedersen BK: **Elevated levels of IL-18 in plasma and skeletal muscle in chronic obstructive pulmonary disease**. *Lung* 2007, **185**(3):161-171.

11. Imaoka H, Hoshino T, Takei S, Kinoshita T, Okamoto M, Kawayama T, Kato S, Iwasaki H, Watanabe K, Aizawa H: **Interleukin-18 production and pulmonary function in COPD**. *Eur Respir J* 2008, **31**(2):287-297.

12. Aldonyte R, Eriksson S, Piitulainen E, Wallmark A, Janciauskiene S: **Analysis of systemic biomarkers in COPD patients**. *COPD* 2004, **1**(2):155-164.

13. Liu SF, Chin CH, Wang CC, Lin MC: **Correlation between serum biomarkers and BODE index in patients with stable COPD**. *Respirology* 2009, **14**(7):999-1004.

14. Capelli A, Di Stefano A, Gnemmi I, Balbo P, Cerutti CG, Balbi B, Lusuardi M, Donner CF: **Increased MCP-1 and MIP-1beta in bronchoalveolar lavage fluid of chronic bronchitics**. *Eur Respir J* 1999, **14**(1):160-165.

15. Traves SL, Culpitt SV, Russell RE, Barnes PJ, Donnelly LE: **Increased levels of the chemokines GROalpha and MCP-1 in sputum samples from patients with COPD**. *Thorax* 2002, **57**(7):590-595.

16. Keatings VM, Barnes PJ: **Granulocyte activation markers in induced sputum: comparison between chronic obstructive pulmonary disease, asthma, and normal subjects**. *Am J Respir Crit Care Med* 1997, **155**(2):449-453.

17. Pesci A, Balbi B, Majori M, Cacciani G, Bertacco S, Alciato P, Donner CF: **Inflammatory cells and mediators in bronchial lavage of patients with chronic obstructive pulmonary disease**. *Eur Respir J* 1998, **12**(2):380-386.

18. Jiang Y, Xiao W, Zhang Y, Xing Y: **Urokinase-type plasminogen activator system and human cationic antimicrobial protein 18 in serum and induced sputum of patients with chronic obstructive pulmonary disease**. *Respirology* 2010, **15**(6):939-946.

19. Ashitani J, Mukae H, Arimura Y, Matsukura S: **Elevated plasma procoagulant and fibrinolytic markers in patients with chronic obstructive pulmonary disease**. *Intern Med* 2002, **41**(3):181-185.

20. Costa C, Rufino R, Traves SL, Lapa ESJR, Barnes PJ, Donnelly LE: **CXCR3 and CCR5 chemokines in induced sputum from patients with COPD**. *Chest* 2008, **133**(1):26-33.

21. Higashimoto Y, Yamagata Y, Taya S, Iwata T, Okada M, Ishiguchi T, Sato H, Itoh H: **Systemic inflammation in chronic obstructive pulmonary disease and asthma: Similarities and differences**. *Respirology* 2008, **13**(1):128-133.

22. Culpitt SV, Rogers DF, Traves SL, Barnes PJ, Donnelly LE: **Sputum matrix metalloproteases: comparison between chronic obstructive pulmonary disease and asthma**. *Respir Med* 2005, **99**(6):703-710.

23. Cheng SL, Wang HC, Yu CJ, Yang PC: **Increased expression of placenta growth factor in COPD**. *Thorax* 2008, **63**(6):500-506.

24. Rovina N, Papapetropoulos A, Kollintza A, Michailidou M, Simoes DC, Roussos C, Gratziou C: **Vascular endothelial growth factor: an angiogenic factor reflecting airway inflammation in healthy smokers and in patients with bronchitis type of chronic obstructive pulmonary disease?** *Respir Res* 2007, **8**:53.

25. Santos S, Peinado VI, Ramirez J, Morales-Blanhir J, Bastos R, Roca J, Rodriguez-Roisin R, Barbera JA: **Enhanced expression of vascular endothelial growth factor in pulmonary arteries of smokers and patients with moderate chronic obstructive pulmonary disease**. *Am J Respir Crit Care Med* 2003, **167**(9):1250-1256.

26. Kythreotis P, Kokkini A, Avgeropoulou S, Hadjioannou A, Anastasakou E, Rasidakis A, Bakakos P: **Plasma leptin and insulin-like growth factor I levels during acute exacerbations of chronic obstructive pulmonary disease**. *BMC Pulm Med* 2009, **9**:11.

27. Itabashi S, Fukushima T, Aikawa T, Yanai M, Sekizawa K, Sasaki H, Takishima T: **Allergic sensitization in elderly patients with chronic obstructive pulmonary disease**. *Respiration* 1990, **57**(6):384-388.

**FIGURE LEGENDS**

**Online Supplement - Figure 1. Associations of biomarkers with history of myocardial event.** Serum levels of creatine kinase (top) and myoglobin (middle) and percent-predicted FEV_1_ (bottom) at baseline for T54 COPD population are shown for patients with a history of myocardial infarction or cardiac ischemia. Bars indicate medians.

**Online Supplement - Figure 2. Supervised clustering within COPD populations.** Hierarchical clustering (Euclidean distance with average linkage) was performed for both patient samples and analytes for the T54 COPD population (left), restricted to the analytes significantly associated with COPD relative to controls. For the BioServe COPD population (right), analytes were ordered in the same order as for the clustered T54 population and hierarchical clustering performed for patient samples only. Serum concentrations were normalized as log_2_ of fold over the geometric mean (G_m_) of the respective COPD population and presented as a heatmap with subjects across x-axis and analytes on y-axis. Smoking status and GOLD stage are represented at bottom.

EGF, epidermal growth factor; RANTES, regulated upon activation, normally T-cell expressed, and secreted; EN-RAGE, extracellular newly identified-receptor for advanced glycation end-binding protein; RA, receptor agonist; IL, interleukin; ENA-78, epithelial-derived neutrophil activating protein-78; PAI-1, plasminogen activating factor-1; MCP-1, monocyte chemoattractant protein-1; MIP-1beta, macrophage inflammatory protein-1beta; TIMP-1, tissue inhibitor of metalloproteinases-1; VEGF, vascular endothelial growth factor; MB, muscle-brain; TNF-RII, tumor necrosis factor-receptor II; IGF-1, insulin-like growth factor; Ig, immunoglobulin.
