# Supplementary material for: Systemic inflammatory profile and response to anti-tumor necrosis factor therapy in chronic obstructive pulmonary disease
Source: Respir Res. 2012 Feb 2;13(1):12. doi: 10.1186/1465-9921-13-12 (PMC3287122; doi:10.1186/1465-9921-13-12)
Supplement: Additional file 2 — Online Supplement - Table S1. Least detectable doses for analytes in Rules-Based Medicine Human MAP v1.6 panel. Analytes' least detectable doses in the Rules-Based Medicine MAP v. 1.6 panel. [file 1465-9921-13-12-S2.DOC]

| **Online Supplement - Table 1. Least detectable doses for analytes in Rules-Based Medicine Human MAP v1.6 panel** | | | | | | | |
| --- | --- | --- | --- | --- | --- | --- | --- |
| **Analyte** |  | **Abbreviation** |  | **Unit** | **LDD^a^** |  | **75th percentile <LDD^b^** |
| Adiponectin |  |  |  | ug/mL | 0.20 |  |  |
| Alpha-1 antitrypsin |  |  |  | mg/mL | 0.011 |  |  |
| Alpha-2 macroglobulin |  |  |  | mg/mL | 0.061 |  |  |
| Alpha-fetoprotein |  |  |  | ng/mL | 0.43 |  |  |
| Apolipoprotein A1 |  |  |  | mg/mL | 0.0066 |  |  |
| Apolipoprotein CIII |  |  |  | ug/mL | 2.7 |  |  |
| Apolipoprotein H |  |  |  | ug/mL | 8.8 |  |  |
| Beta-2 microglobulin |  |  |  | ug/mL | 0.013 |  |  |
| Brain-derived neurotrophic factor |  | BDNF |  | ng/mL | 0.029 |  |  |
| C-reactive protein |  | CRP |  | ug/mL | 0.0015 |  |  |
| Calcitonin |  |  |  | pg/mL | 6.0 |  | <LDD |
| Cancer antigen 125 |  | CA125 |  | U/mL | 4.2 |  |  |
| Cancer antigen 19-9 |  | CA19-9 |  | U/mL | 0.25 |  |  |
| Carcinoembryonic antigen |  | CEA |  | ng/mL | 0.84 |  |  |
| CD40 |  |  |  | ng/mL | 0.021 |  |  |
| CD40 ligand |  | CD40L |  | ng/mL | 0.020 |  |  |
| Complement 3 |  |  |  | mg/mL | 0.0053 |  |  |
| Creatine kinase-MB |  |  |  | ng/mL | 0.42 |  |  |
| Epidermal growth factor |  | EGF |  | pg/mL | 7.4 |  |  |
| Epithelial-derived neutrophil activating protein-78 |  | ENA-78 |  | ng/mL | 0.076 |  |  |
| Endothelin-1 |  |  |  | pg/mL | 7.2 |  | <LDD |
| EN-RAGE (S100A2) |  |  |  | ng/mL | 0.25 |  |  |
| Eotaxin |  |  |  | pg/mL | 41 |  |  |
| Erythropoietin |  |  |  | pg/mL | 166 |  | <LDD |
| Factor VII |  |  |  | ng/mL | 1.0 |  |  |
| Fatty acid binding protein |  |  |  | ng/mL | 3.0 |  | dichotomize^c^ |
| Ferritin |  |  |  | ng/mL | 1.4 |  |  |
| Fibroblast growth factor, basic |  | FGF basic |  | pg/mL | 98 |  |  |
| Fibrinogen |  |  |  | mg/mL | 0.0098 |  | <LDD |
| Granulocyte-colony stimulating factor |  | G-CSF |  | pg/mL | 5.0 |  |  |
| Glutathione-S-transferase |  | GST |  | ng/mL | 0.40 |  |  |
| Granulocytemacrophage-colony stimulating factor |  | GM-CSF |  | pg/mL | 57 |  | <LDD |
| Growth hormone |  |  |  | ng/mL | 0.13 |  |  |
| Haptoglobin |  |  |  | mg/mL | 0.025 |  |  |
| Intercellular adhesion molecule-1 |  | ICAM-1 |  | ng/mL | 3.2 |  |  |
| Interferon-gamma |  | IFN-gamma |  | pg/mL | 4.6 |  | dichotomize^c^ |
| Immunoglobulin A |  | IgA |  | mg/mL | 0.0084 |  |  |
| Immunoglobulin E |  | IgE |  | ng/mL | 14 |  |  |
| Insulin-like growth factor-1 |  | IGF-1 |  | ng/mL | 4.0 |  |  |
| Immunoglobulin M |  | IgM |  | mg/mL | 0.015 |  |  |
| Interleukin-10 |  | IL-10 |  | pg/mL | 15 |  | <LDD |
| Interleukin-12p40 |  | IL-12p40 |  | ng/mL | 1.2 |  | <LDD |
| Interleukin-12p70 |  | IL-12p70 |  | pg/mL | 94 |  | <LDD |
| Interleukin-13 |  | IL-13 |  | pg/mL | 57 |  |  |
| Interleukin-15 |  | IL-15 |  | ng/mL | 1.3 |  | <LDD |
| Interleukin-16 |  | IL-16 |  | pg/mL | 66 |  |  |
| Interleukin-17 |  | IL-17 |  | pg/mL | 2.7 |  |  |
| Interleukin-18 |  | IL-18 |  | pg/mL | 54 |  |  |
| Interleukin-1alpha |  | IL-1alpha |  | ng/mL | 0.16 |  | dichotomize^c^ |
| Interleukin-1beta |  | IL-1beta |  | pg/mL | 1.5 |  | <LDD |
| Interleukin-1RA |  | IL-1RA |  | pg/mL | 15 |  |  |
| Interleukin-2 |  | IL-2 |  | pg/mL | 60 |  | <LDD |
| Interleukin-23 |  | IL-23 |  | ng/mL | <0.67 |  | <LDD |
| Interleukin-3 |  | IL-3 |  | ng/mL | 0.17 |  | <LDD |
| Interleukin-4 |  | IL-4 |  | pg/mL | 104 |  | dichotomize^c^ |
| Interleukin-5 |  | IL-5 |  | pg/mL | 33 |  | <LDD |
| Interleukin-6 |  | IL-6 |  | pg/mL | 12 |  | <LDD |
| Interleukin-7 |  | IL-7 |  | pg/mL | 53 |  |  |
| Interleukin-8 |  | IL-8 |  | pg/mL | 3.5 |  |  |
| Insulin |  |  |  | uIU/mL | 0.86 |  |  |
| Leptin |  |  |  | ng/mL | 0.10 |  |  |
| Lipoprotein (a) |  |  |  | ug/mL | 3.7 |  |  |
| Lymphotactin |  |  |  | ng/mL | 0.38 |  | <LDD |
| Monocyte chemoattractant protein-1 |  | MCP-1 |  | pg/mL | 52 |  |  |
| Monocyte-derived chemokine |  | MDC |  | pg/mL | 14 |  |  |
| Macrophage inflammatory protein-1alpha |  | MIP-1alpha |  | pg/mL | 13 |  |  |
| Macrophage inflammatory protein-1beta |  | MIP-1beta |  | pg/mL | 38 |  |  |
| Matrix metalloproteinase-2 |  | MMP-2 |  | ng/mL | 150 |  | <LDD |
| Matrix metalloproteinase-3 |  | MMP-3 |  | ng/mL | 0.20 |  |  |
| Matrix metalloproteinase-9 |  | MMP-9 |  | ng/mL | 37 |  | <LDD |
| Myeloperoxidase |  |  |  | ng/mL | 68 |  |  |
| Myoglobin |  |  |  | ng/mL | 1.1 |  |  |
| Plasminogen activating factor-1 |  | PAI-1 |  | ng/mL | 0.90 |  |  |
| Pregnancy-associated plasma protein-A |  | PAPP-A |  | mIU/mL | 0.037 |  | <LDD |
| Prostate specific antigen, free |  |  |  | ng/mL | 0.023 |  |  |
| Prostatic acid phosphatase |  |  |  | ng/mL | 0.034 |  |  |
| Regulated upon activation, normally T cell-expressed, and secreted |  | RANTES |  | ng/mL | 0.048 |  |  |
| Serum amyloid P |  |  |  | ug/mL | 0.058 |  |  |
| Glutamic oxaloacetic transaminase |  | SGOT |  | ug/mL | 3.7 |  |  |
| Sex hormone-binding globulin |  | SHBG |  | nmol/L | 1.3 |  |  |
| Stem cell factor |  |  |  | pg/mL | 56 |  |  |
| Thrombopoietin |  |  |  | ng/mL | 3.2 |  |  |
| Thyroid stimulating hormone |  |  |  | uIU/mL | 0.028 |  |  |
| Thyroxine binding globulin |  |  |  | ug/mL | 0.34 |  |  |
| Tissue inhibitor of metalloproteinases-1 |  | TIMP-1 |  | ng/mL | 8.4 |  |  |
| Tissue factor |  |  |  | ng/mL | 0.84 |  | <LDD |
| Tumor necrosis factor-receptor II |  | TNF-RII |  | ng/mL | 0.13 |  |  |
| Tumor necrosis factor-alpha |  | TNF-alpha |  | pg/mL | 4.0 |  |  |
| Tumor necrosis factor-beta |  | TNF-beta |  | pg/mL | 46 |  | <LDD |
| Vascular cellular adhesion molecule-1 |  | VCAM-1 |  | ng/mL | 2.6 |  |  |
| Vascular endothelial growth factor |  | VEGF |  | pg/mL | 7.5 |  |  |
| von Willebrand Factor |  |  |  | ug/mL | 0.40 |  |  |

^a^LDD, defined as the back-calculated concentration for the mean plus 3 standard deviations of luminescence values of 20 blank readings.

^b^Analytes marked "<LDD" were below LDD for >75% of samples in each COPD and control population. These analytes were excluded from further statistical analyses of the respective sets of data.

^c^Analytes marked "dichotomize" were below LDD for >75% of samples in the COPD (or control) population and between 50-75% of samples in the control (or COPD) population. These analytes were dichotomized as being above or below LDD and treated as binary variables for statistical analyses.

COPD, chronic obstructive pulmonary disease; EN-RAGE, extracellular newly identified-receptor for advanced glycation end-binding protein; LDD, least detectable dose; MB, muscle-brain.
