# Supplementary material for: Systemic inflammatory profile and response to anti-tumor necrosis factor therapy in chronic obstructive pulmonary disease
Source: Respir Res. 2012 Feb 2;13(1):12. doi: 10.1186/1465-9921-13-12 (PMC3287122; doi:10.1186/1465-9921-13-12)
Supplement: Additional file 3 — Online Supplement- Table S2. Analytes demonstrating potential batch effects. Analytes' demonstrating potential batch effects. [file 1465-9921-13-12-S3.DOC]

| **Online Supplement - Table 2. Analytes demonstrating potential batch effects** | | | |
| --- | --- | --- | --- |
| Analyte |  | ±fold (Ctr2/Ctr1) | p-value |
| Cancer antigen 125 (CA125) |  | 2.4 | 2.0 x 10^-7^ |
| Cancer antigen 19-9 (CA19-9) |  | 0.6 | 0.029 |
| Glutathione-S-transferase (GST) |  | 5.00 | <10^-9^ |
| Interleukin-17 (IL-17) |  | -0.7 | 1 x 10^-9^ |
| Serum glutamic-oxaloacetic |  | -0.9 | 3 x 10^-8^ |
| transaminase (SGOT) |  |  |  |
|  |  |  |  |
| Signed-fold (±fold)=median of Ctr1 population/median of Ctr2 population. | | | |
| P-values from Mann-Whitney U test for Ctr1 vs Ctr2 populations. | | | |
