# Supplementary material for: Systemic inflammatory profile and response to anti-tumor necrosis factor therapy in chronic obstructive pulmonary disease
Source: Respir Res. 2012 Feb 2;13(1):12. doi: 10.1186/1465-9921-13-12 (PMC3287122; doi:10.1186/1465-9921-13-12)
Supplement: Additional file 4 — Online Supplement - Table S3. Gender- and race-restricted analyses for associations with COPD. COPD associations with gender- and race-restricted analyses. [file 1465-9921-13-12-S4.DOC]

| **Online Supplement - Table 3. Gender- and race-restricted analyses for associations with COPD** | | | | | | |  |  |  |  |  |  |
| --- | --- | --- | --- | --- | --- | --- | --- | --- | --- | --- | --- | --- |
| **Analyte^a^** |  | **Black^b^** | |  | **Caucasian** | |  | **Males** | |  | **Females** | |
|  |  | **fold/**  **control^c^** | **FDR** |  | **fold/ control** | **FDR** |  | **fold/ control** | **FDR** |  | **fold/ control** | **FDR** |
| CD40 ligand |  | 122.33 | <10^-8^ |  | 168.50 | <10^-8^ |  | 142.00 | <10^-8^ |  | 180.00 | <10^-8^ |
| Epidermal growth factor |  | 50.49 | <10^-8^ |  | 105.54 | <10^-8^ |  | 29.37 | <10^-8^ |  | 47.55 | <10^-8^ |
| Brain-derived neurotrophic factor |  | 46.53 | <10^-8^ |  | 55.72 | <10^-8^ |  | 53.36 | <10^-8^ |  | 47.65 | <10^-8^ |
| Regulated upon activation, normally T-cell expressed, and secreted |  | 21.94 | <10^-8^ |  | 15.83 | <10^-8^ |  | 12.92 | <10^-8^ |  | 15.09 | <10^-8^ |
| Myeloperoxidase |  | 7.64 | <10^-8^ |  | 17.22 | <10^-8^ |  | 8.88 | <10^-8^ |  | 12.90 | <10^-8^ |
| Eotaxin |  | 4.25 | 0.0027 |  | 9.76 | <10^-8^ |  | 9.32 | <10^-8^ |  | 9.27 | <10^-8^ |
| EN-RAGE |  | 4.38 | 4.4 x 10^-5^ |  | 4.37 | <10^-8^ |  | 3.93 | <10^-8^ |  | 5.15 | <10^-8^ |
| Ferritin |  | 5.34 | 6.1 x 10^-6^ |  | 4.46 | <10^-8^ |  | 6.38 | <10^-8^ |  | 4.72 | <10^-8^ |
| Interleukin-1RA |  | 4.34 | 6.1 x 10^-6^ |  | 4.24 | <10^-8^ |  | 4.43 | <10^-8^ |  | 4.38 | <10^-8^ |
| Epithelial-derived neutrophil activating protein-78 |  | 12.85 | <10^-8^ |  | 7.50 | <10^-8^ |  | 5.79 | <10^-8^ |  | 5.75 | <10^-8^ |
| Plasminogen activating factor-1 |  | 5.37 | <10^-8^ |  | 2.96 | <10^-8^ |  | 5.22 | <10^-8^ |  | 4.48 | <10^-8^ |
| Monocyte chemoattractant protein-1 |  | 1.95 | <10^-8^ |  | 4.93 | <10^-8^ |  | 3.44 | <10^-8^ |  | 4.63 | <10^-8^ |
| Macrophage inflammatory protein-1beta |  | 4.54 | <10^-8^ |  | 3.23 | <10^-8^ |  | 2.92 | <10^-8^ |  | 3.35 | <10^-8^ |
| Thrombopoietin |  | 2.98 | <10^-8^ |  | 2.96 | <10^-8^ |  | 2.89 | <10^-8^ |  | 3.03 | <10^-8^ |
| Tissue inhibitor of metalloproteinases-1 |  | 2.93 | <10^-8^ |  | 2.85 | <10^-8^ |  | 2.91 | <10^-8^ |  | 3.13 | <10^-8^ |
| Interleukin-16 |  | 2.05 | <10^-8^ |  | 1.76 | <10^-8^ |  | 2.13 | <10^-8^ |  | 2.06 | <10^-8^ |
| Vascular endothelial growth factor |  | 3.22 | <10^-8^ |  | 2.29 | <10^-8^ |  | 2.44 | <10^-8^ |  | 2.63 | <10^-8^ |
| Cancer antigen 19-9 |  | 1.23 | **0.53** |  | 1.22 | **0.40** |  | 1.78 | 0.00036 |  | 2.10 | 0.00016 |
| CD40 |  | 2.38 | <10^-8^ |  | 1.97 | <10^-8^ |  | 2.01 | <10^-8^ |  | 2.53 | <10^-8^ |
| Creatine kinase-MB |  | 2.09 | 0.014 |  | 2.23 | 2.2 x 10^-6^ |  | 2.30 | <10^-8^ |  | 2.05 | <10^-8^ |
| C-reactive protein |  | 2.50 | 0.012 |  | 2.03 | **0.078** |  | 2.35 | <10^-8^ |  | 1.62 | **0.054** |
| Myoglobin |  | 1.65 | 0.014 |  | 1.70 | <10^-8^ |  | 1.82 | <10^-8^ |  | 2.19 | <10^-8^ |
| Stem cell factor |  | 2.73 | <10^-8^ |  | 1.96 | <10^-8^ |  | 1.85 | <10^-8^ |  | 2.24 | <10^-8^ |
| Interleukin-18 |  | 1.90 | <10^-8^ |  | 1.44 | 3.1 x 10^-5^ |  | 1.78 | <10^-8^ |  | 1.71 | <10^-8^ |
| Tumor necrosis factor-receptor II |  | 1.50 | <10^-8^ |  | 1.47 | <10^-8^ |  | 1.73 | <10^-8^ |  | 1.49 | <10^-8^ |
|  |  |  |  |  |  |  |  |  |  |  |  |  |
| Insulin-like growth factor-1 |  | -4.09 | 5.583E-05 |  | -4.42 | 0.00019 |  | -6.45 | <10^-8^ |  | -4.68 | <10^-8^ |
| Immunoglobulin E |  | -1.28 | **0.13** |  | -2.20 | 0.033 |  | -2.29 | 0.00029 |  | -3.90 | 1.8 x 10^-6^ |

^a^Baseline levels of analytes reported in Table 2 to be associated with COPD vs control, in the order presented in Table 2.

^b^COPD vs control associations were evaluated within the indicated racial and gender subpopulations. The subpopulation sizes were (COPD/control): black, 24/66; Caucasian, 366/27; Males, 225/84, Females, 169/62.

^c^FDR and signed-fold levels in COPD over controls (fold/control) are provided for each subpopulation. FDR>0.05 are shown in bold.

COPD, chronic obstructive pulmonary disease; FDR, false discovery rate; MB, muscle-brain; RA, receptor agonist.
