# Supplementary material for: Systemic inflammatory profile and response to anti-tumor necrosis factor therapy in chronic obstructive pulmonary disease
Source: Respir Res. 2012 Feb 2;13(1):12. doi: 10.1186/1465-9921-13-12 (PMC3287122; doi:10.1186/1465-9921-13-12)
Supplement: Additional file 5 — Online Supplement - Table S4. Associations of baseline analyte levels with smoking status. Baseline analyte levels and their associations with smoking status. [file 1465-9921-13-12-S5.DOC]

| **Online Supplement - Table 4. Associations of baseline analyte levels with smoking status** | | | | | |  |  |  |  |
| --- | --- | --- | --- | --- | --- | --- | --- | --- | --- |
| **Analyte** |  | **COPD T54^a^** |  |  | **COPD BioServe** |  |  | **Healthy controls (Ctr1+Ctr2)** |  |
|  |  | **Current/Ex-smoker^b^** | **FDR^c^** |  | **Current/Ex-smoker** | **FDR** |  | **Current/Non-smoker** | **FDR** |
| Carcinoembryonic antigen |  | 1.93 | <10^-8^ |  | 1.59 | 0.00047 |  | 1.61 | 0.0087 |
| Monocyte-derived chemokine |  | 1.29 | <10^-8^ |  | 1.25 | 0.032 |  | 1.03 | 0.70 |
| beta2 microglobulin |  | -1.26 | <10^-8^ |  | -1.08 | 0.37 |  | 1.00 | 0.88 |
| Vascular cellular adhesion molecule-1 |  | -1.18 | 0.00028 |  | -1.09 | 0.66 |  | 0.94 | 0.88 |
| Intercellular adhesion molecule-1 |  | 1.15 | 0.00029 |  | 1.11 | 0.36 |  | 1.06 | 0.70 |
| Matrix metalloproteinase-3 |  | -1.25 | 0.00096 |  | -1.51 | 0.35 |  | 1.06 | 0.76 |
| Myoglobin |  | -1.22 | 0.0017 |  | -1.32 | 0.35 |  | 1.18 | 0.76 |
| von Willebrand factor |  | -1.28 | 0.0017 |  | -1.03 | 0.85 |  | 1.08 | 0.78 |
| Tumor necrosis factor-receptor II |  | -1.15 | 0.0029 |  | -1.01 | 0.80 |  | 1.00 | 0.88 |
| Apolipoprotein H |  | -1.11 | 0.011 |  | 1.03 | 0.99 |  | 1.11 | 0.15 |
| Haptoglobin |  | 1.34 | 0.013 |  | 1.07 | 0.85 |  | 1.69 | 0.70 |
| Adiponectin |  | -1.34 | 0.026 |  | 1.08 | 0.80 |  | 1.09 | 0.90 |
| Leptin |  | -1.45 | 0.026 |  | -1.75 | 0.35 |  | -3.85 | 0.0079^d^ |
| Plasminogen activating factor-1 |  | 1.14 | 0.059 |  | 1.08 | 0.36 |  | 0.71 | 0.88 |
| Apolipoprotein A1 |  | -1.13 | 0.082 |  | -1.03 | 0.94 |  | 1.06 | 0.76 |
| Eotaxin |  | 1.30 | 0.082 |  | -1.07 | 0.37 |  | 1.00 | 0.78 |
| Thyroid stimulating hormone |  | -1.25 | 0.14 |  | -1.23 | 0.36 |  | 1.05 | 0.90 |
| Interleukin-16 |  | -1.15 | 0.20 |  | 1.27 | 0.37 |  | 0.94 | 0.88 |
| Brain-derived neurotrophic factor |  | 1.11 | 0.20 |  | 1.11 | 0.37 |  | 1.04 | 0.88 |
| Cancer antigen 125 |  | -1.19 | 0.20 |  | -1.16 | 0.99 |  | 1.19 | 0.99 |
| Fibroblast growth factor, basic |  | 1.09 | 0.39 |  | -1.03 | 0.65 |  | 1.31 | 0.76 |
| CD40 ligand |  | 1.22 | 0.46 |  | 1.22 | 0.73 |  | 1.00 | 0.76 |
| Immunoglobulin A |  | -1.12 | 0.46 |  | 1.02 | 0.94 |  | 0.94 | 0.99 |
| Interleukin-8 |  | 1.24 | 0.46 |  | -1.07 | 0.99 |  | 1.91 | 0.31 |
| Prostate specific antigen (free) |  | -1.81 | 0.46 |  | 1.93 | 0.89 |  | 11.76 | 0.0087^d^ |
| Regulated upon activation, normally T-cell expressed, and secreted |  | 1.08 | 0.46 |  | 1.30 | 0.37 |  | 1.15 | 0.90 |
| Apolipoprotein CIII |  | -1.05 | 0.47 |  | -1.19 | 0.37 |  | 1.16 | 0.76 |
| Stem cell factor |  | -1.05 | 0.47 |  | 1.06 | 0.73 |  | 1.30 | 0.43 |
| Complement 3 |  | -1.04 | 0.54 |  | -1.03 | 0.80 |  | 0.91 | 0.63 |
| Creatine kinase-MB |  | -1.08 | 0.54 |  | -1.05 | 0.85 |  | 1.00 | 0.99 |
| Sex hormone-binding globulin |  | -1.08 | 0.54 |  | -1.05 | 0.37 |  | 0.92 | 0.90 |
| Epidermal growth factor |  | 1.35 | 0.56 |  | 1.22 | 0.35 |  | 1.06 | 0.99 |
| alpha2 macroglobulin |  | -1.03 | 0.56 |  | -1.08 | 0.89 |  | 1.08 | 0.88 |
| Granulocyte-colony stimulating factor |  | 1.08 | 0.56 |  | 1.00 | 0.80 |  | 0.88 | 0.52 |
| Epithelial-derived neutrophil activating protein-78 |  | 1.09 | 0.59 |  | 1.37 | 0.35 |  | 0.81 | 0.76 |
| Interleukin-18 |  | -1.06 | 0.59 |  | 1.19 | 0.36 |  | 0.94 | 0.88 |
| C-reactive protein |  | -1.19 | 0.67 |  | -1.24 | 0.59 |  | 0.83 | 0.70 |
| Myeloperoxidase |  | 1.20 | 0.67 |  | 1.49 | 0.40 |  | 1.03 | 0.90 |
| Tissue inhibitor of metalloproteinases-1 |  | -1.02 | 0.67 |  | -1.03 | 0.89 |  | 1.02 | 0.99 |
| Immunoglobulin E |  | -1.13 | 0.73 |  | 1.35 | 0.80 |  | 1.21 | 0.99 |
| Interleukin-1RA |  | -1.07 | 0.73 |  | 1.72 | 0.37 |  | -1.89 | 0.0036 |
| Insulin |  | 1.21 | 0.73 |  | -1.44 | 0.37 |  | -1.62 | 0.63 |
| Macrophage inflammatory protein-1beta |  | -1.08 | 0.73 |  | -1.06 | 0.94 |  | 1.06 | 0.88 |
| Ferritin |  | -1.16 | 0.83 |  | -1.03 | 0.85 |  | 1.49 | 0.70 |
| Tumor necrosis factor-alpha |  | 1.02 | 0.83 |  | -1.04 | 0.94 |  | 1.00 | 0.99 |
| Interleukin-13 |  | 1.00 | 0.85 |  | 1.03 | 0.89 |  | 1.00 | 0.99 |
| Interleukin-17 |  | 1.05 | 0.85 |  | 1.04 | 0.49 |  | 1.09 | 0.63 |
| Interleukin-7 |  | 1.05 | 0.85 |  | 1.04 | 0.80 |  | 1.10 | 0.99 |
| Lipoprotein A |  | 1.17 | 0.85 |  | 1.92 | 0.41 |  | 0.89 | 0.99 |
| Monocyte chemoattractant protein-1 |  | -1.01 | 0.85 |  | -1.21 | 0.37 |  | 1.13 | 0.76 |
| Prostatic acid phosphatase |  | -1.01 | 0.85 |  | 1.14 | 0.36 |  | 1.28 | 0.15 |
| Sex hormone-binding globulin |  | 1.09 | 0.85 |  | 1.15 | 0.80 |  | 1.10 | 0.76 |
| Vascular endothelial growth factor |  | 1.02 | 0.85 |  | 1.05 | 0.89 |  | 1.01 | 0.90 |
| Alpha1-antitrypsin |  | 1.05 | 0.86 |  | 1.08 | 0.79 |  | 1.00 | 0.88 |
| CD40 |  | -1.05 | 0.86 |  | 1.07 | 0.80 |  | 1.15 | 0.43 |
| Factor VII |  | -1.10 | 0.86 |  | -1.06 | 0.53 |  | 1.02 | 0.99 |
| Glutathione-S-transferase |  | 1.01 | 0.86 |  | -1.14 | 0.94 |  | 0.95 | 0.78 |
| Serum amyloid P |  | -1.03 | 0.86 |  | -1.06 | 1.00 |  | 1.07 | 0.99 |
| Thyroxine binding globulin |  | 1.02 | 0.86 |  | -1.00 | 0.94 |  | 0.90 | 0.78 |
| Alpha-fetoprotein |  | 1.01 | 0.89 |  | -1.05 | 0.94 |  | 1.23 | 0.58 |
| Immunoglobulin M |  | 1.06 | 0.98 |  | -1.05 | 0.94 |  | 0.80 | 0.25 |
| Cancer antigen 19-9 |  | 1.01 | 0.98 |  | -1.07 | 0.49 |  | 1.32 | 0.76 |
| EN-RAGE |  | 1.10 | 0.98 |  | 1.27 | 0.40 |  | 1.17 | 0.70 |
| Growth hormone |  | -1.02 | 0.98 |  | 1.36 | 0.93 |  | -2.52 | 0.76 |
| Insulin-like growth hormone-1 |  | -1.01 | 0.98 |  | 1.06 | 0.94 |  | 0.94 | 0.76 |
| Thrombopoietin |  | 1.01 | 0.98 |  | 1.02 | 0.44 |  | 1.01 | 0.97 |
| Macrophage inflammatory protein-1alpha |  | 1.03 | 1.00 |  | -1.01 | 0.94 |  | 1.19 | 0.52 |

^a^Baseline levels of analytes were compared between current smokers and either ex-smokers (for COPD) or non-smokers (for controls). Ctr1 and Ctr2 control populations were combined for this analysis. The sample sizes were (current/ex-smoker, non-smoker): T54, 104/130; BioServe, 50/100; Ctr1+Ctr2, 69/40.

^b^Signed-fold=median in COPD/median in healthy control population. When median expression in COPD population was lower than that in controls, the opposite of the reciprocal is reported.

^c^FDR (calculated from Mann-Whitney p-values) for current smoker vs ex-smoker or non-smoker subpopulations.

^d^When adjusted for gender, the significance of association became non-significant.

COPD, chronic obstructive pulmonary disease; FDR, false discovery rate; MB, muscle-brain; RA, receptor agonist.
