# Supplementary figures and images for: Systemic inflammatory profile and response to anti-tumor necrosis factor therapy in chronic obstructive pulmonary disease
Source: Respir Res. 2012 Feb 2;13(1):12. doi: 10.1186/1465-9921-13-12 (PMC3287122; doi:10.1186/1465-9921-13-12)

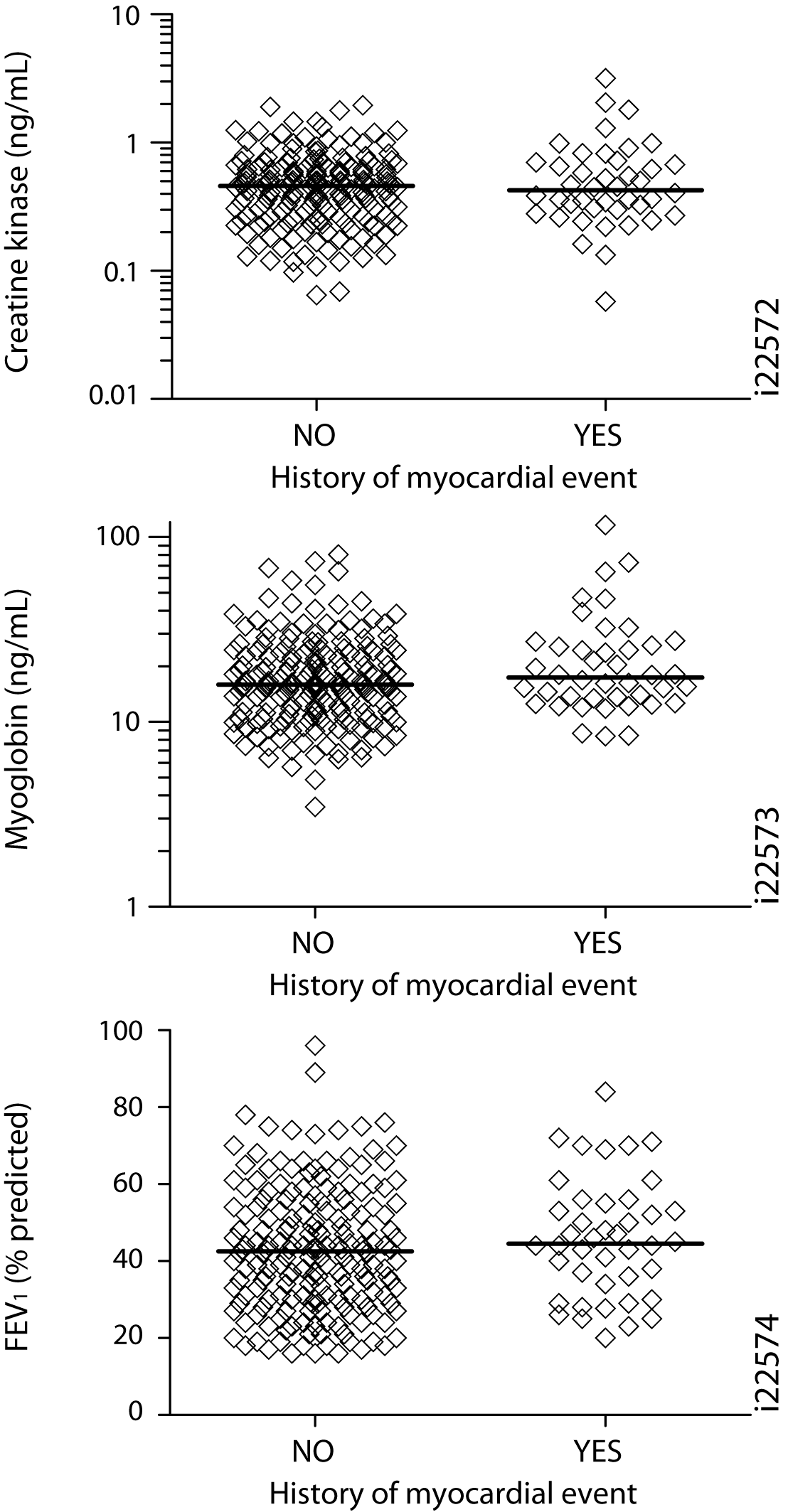

Supplement: Additional file 6 — Online Supplement - Figure S1. Associations of biomarkers with history of myocardial event. Three graphs show serum levels of patients with a history of myocardial infarction or cardiac ischemia. [file 1465-9921-13-12-S6.TIFF]
