# Supplementary material for: Systemic inflammatory profile and response to anti-tumor necrosis factor therapy in chronic obstructive pulmonary disease
Source: Respir Res. 2012 Feb 2;13(1):12. doi: 10.1186/1465-9921-13-12 (PMC3287122; doi:10.1186/1465-9921-13-12)
Supplement: Additional file 7 — Online Supplement - Figure S2. Supervised clustering within COPD populations. Heatmap of supervised clustering within populations with COPD. [file 1465-9921-13-12-S7.DOCX]

**Online Supplement - Figure 2**
