# Supplementary material for: Systemic inflammatory profile and response to anti-tumor necrosis factor therapy in chronic obstructive pulmonary disease
Source: Respir Res. 2012 Feb 2;13(1):12. doi: 10.1186/1465-9921-13-12 (PMC3287122; doi:10.1186/1465-9921-13-12)
Supplement: Additional file 8 — Online Supplement - Table S5. Previous reports for COPD-associated analytes identified herein. Compares previously reported and identified COPD-associated analytes. [file 1465-9921-13-12-S8.DOC]

| **Online Supplement - Table 5. Previous reports for COPD-associated analytes identified herein** | | | | |  |  |  |
| --- | --- | --- | --- | --- | --- | --- | --- |
|  |  |  |  |  |  |  |  |
| **Analyte^a^** |  | **Serum** | | |  | **Airway (sputum, biopsy, BAL)** | |
|  |  | **Pinto-Plata^b^** | **Aaron^c^** | **Others studies^d^** |  | **Aaron^c^** | **Others studies^d^** |
| Brain-derived neurotrophic factor |  | Sll | - |  |  | - |  |
| C-reactive protein |  | - | S | E[[1, 2]] |  | <LDD |  |
| Cancer antigen 19-9 |  | - | - |  |  | - |  |
| CD40 |  | NS | - |  |  | - | e |
| CD40 ligand |  | - | - |  |  | - |  |
| Creatine kinase-MB |  | - | - |  |  | - | e |
| Epidermal growth factor |  | P | - |  |  | - | E[[3]] |
| Epithelial-derived neutrophil activating protein-78 |  | NS | NS |  |  | NS | E[[4]] |
| EN-RAGE |  | - | - |  |  | - |  |
| Eotaxin |  | P | - | C[[5, 6]] | z | - | C[[7-9]] |
| Ferritin |  | - | - |  |  | - |  |
| Interleukin-16 |  | NS | - |  |  | - |  |
| Interleukin-18 |  | NS | NS | E[[10, 11]] |  | NS | E[[11]] |
| Interleukin-1RA |  | P | <LDD |  |  | NS |  |
| Monocyte chemoattractant protein-1 |  | P | NS | C[[6, 12, 13]] |  | S | C[[6],[14, 15]] |
| Macrophage inflammatory protein-1beta |  | NS | - | E[[6]] |  | - | E[[14]] |
| Myeloperoxidase |  | - | S |  |  | <LDD | E[[16, 17]] |
| Myoglobin |  | - | - |  |  | - |  |
| Plasminogen activating factor-1 |  | - | - | E[[18, 19]] |  | - | E[[18]] |
| Regulated upon activation, normally T-cell expressed, and secreted |  | NS | - |  |  | - | E[[20]] |
| Stem cell factor |  | NS | - |  |  | - |  |
| Thrombopoietin |  | - | - |  |  | - |  |
| Tissue inhibitor of metalloproteinases-1 |  | S | NS | E[[21]] |  | S | E[[22]] |
| Tumor necrosis factor-receptor II |  | - | NS |  |  | S |  |
| Vascular endothelial growth factor |  | P | S | E[[12]] |  | S | C[[23-25]] |
|  |  |  |  |  |  |  |  |
| **COPD < Control** |  |  |  |  |  |  |  |
| Insulin-like growth factor-1 |  | - | - | D[[26]] |  | - |  |
| Immunoglobulin E |  | - | - | C[[27]] |  | - |  |

^a^Analytes significantly associated with COPD in current study as described in Table 2.

^b^Pinto-Plata et al.,[28] using microarray platform technology to analyze 143 serum proteins for COPD vs healthy controls. MMP-9 was also significantly elevated in serum of COPD patients in this study.

^c^Aaron et al.,[29] using multiplex immunoassays to analyze 19 serum and 22 induced sputum proteins for COPD vs healthy controls or smoker controls. IL-6 and IL-8 were also significantly elevated in induced sputum from COPD patients in this study.

^d^PubMed searches were performed using COPD and the indicated analyte (using various aliases as appropriate) as search terms. Publications that may have included the indicated analyte but did not include the analyte among searchable terms might not have been identified. In cases where multiple publications support differential expression, the number of such cited may have been limited and is not intended to be exhaustive.

^e^CD40 was reported to be elevated in adipose tissue[30] and creatine kinase elevated in muscle tissue.[31]

COPD, chronic obstructive pulmonary disease; C, conflicting reports; D, significantly decreased (fold-change not considered); E, significantly elevated (fold-change not considered); EN-RAGE, extracellular newly identified-receptor for advanced glycation end-binding protein; MB, muscle-brain; NS, not significant; P, pass significance cutoff (Aaron, p<0.05; Pinto-Plata, FDR<0.05) but fold/control <1.5; S, significant p (Aaron) or FDR (Pinto-Plata) <0.05 and fold/control >1.5; <LDD, majority of measurements in both groups below least detectable dose/lower limit of quantitation; RA, receptor agonist; -, not included in multiplex analysis.
